# IM-ZDD: A Feature-Enhanced Inverse Mapping Framework for Zero-Day Attack Detection in Internet of Vehicles

**DOI:** 10.3390/s25196197

**Published:** 2025-10-06

**Authors:** Tao Chen, Gongyu Zhang, Bingfeng Xu

**Affiliations:** College of Information Science and Technology & College of Artificial Intelligence, Nanjing Forestry University, Nanjing 210037, China; tchen@njfu.edu.cn (T.C.); 2211605124@njfu.edu.cn (G.Z.)

**Keywords:** internet of vehicles, zero-day attack, conditional generative adversarial network, inverse mapping, anomaly detection

## Abstract

In the Internet of Vehicles (IoV), zero-day attacks pose a significant security threat. These attacks are characterized by unknown patterns and limited sample availability. Traditional anomaly detection methods often fail because they rely on oversimplified assumptions, hindering their ability to model complex normal IoV behavior. This limitation results in low detection accuracy and high false alarm rates. To overcome these challenges, we propose a novel zero-day attack detection framework based on Feature-Enhanced Inverse Mapping (IM-ZDD). The framework introduces a two-stage process. In the first stage, a feature enhancement module mitigates data scarcity by employing an innovative multi-generator, multi-discriminator Conditional GAN (CGAN) with dynamic focusing loss to generate a large-scale, high-quality synthetic normal dataset characterized by sharply defined feature boundaries. In the second stage, a learning-based inverse mapping module is trained exclusively on this synthetic data. Through adversarial training, the module learns a precise inverse mapping function, thereby establishing a compact and expressive representation of normal behavior. During detection, samples that cannot be effectively mapped are identified as attacks. Experimental results on the F2MD platform show IM-ZDD achieves superior accuracy and a low false alarm rate, yielding an average AUC of 98.25% and F1-Score of 96.41%, surpassing state-of-the-art methods by up to 4.4 and 10.8 percentage points. Moreover, with a median detection latency of only 3 ms, the framework meets real-time requirements, providing a robust solution for zero-day attack detection in data-scarce IoV environments.

## 1. Introduction

The Internet of Vehicles (IoV) facilitates seamless integration and real-time information exchange among vehicles, infrastructure, pedestrians, and the cloud via Vehicle-to-Everything (V2X) communication. It has found widespread application in fields such as autonomous driving and intelligent traffic management, significantly enhancing traffic efficiency and operational safety [1]. However, this high degree of openness and connectivity also exposes vehicle control systems to growing cybersecurity threats, making them a prime target for network attacks [2]. Among these threats, zero-day attacks represent one of the most formidable challenges. By exploiting previously unknown vulnerabilities, such attacks can bypass traditional signature-based defense systems. They often manifest as subtle, unpredictable anomalies within communication or behavioral data. Yet their impact can be catastrophic, ranging from loss of vehicle control and traffic paralysis to even life-threatening situations [3]. For example, recent top-tier security competitions like Pwn2Own Automotive 2024 have repeatedly demonstrated that attackers can successfully penetrate and remotely control core components of modern vehicles, including Teslas, by chaining together multiple undisclosed vulnerabilities [4,5]. Consequently, the development of a proactive and precise mechanism for identifying such unknown threats has become fundamental to ensuring the safety and resilience of intelligent transportation systems.

To counter zero-day attacks in IoV, existing research primarily adopts two main approaches: knowledge transfer-based methods [6] and anomaly detection-based methods [7]. Knowledge transfer approaches aim to leverage insights learned from known attack types to detect previously unseen threats [8,9]. These methods rest on the assumption that different types of attacks share fundamental similarities in their principles. However, the distinct physical constraints and dynamic communication topologies inherent to IoV environments differ markedly from those of conventional network scenarios. This discrepancy significantly weakens the relevance of cross-domain or cross-type knowledge. It often results in negative transfer, where irrelevant patterns are mistakenly applied, thus undermining detection accuracy in the target domain. In contrast, anomaly detection methods focus on identifying behaviors that deviate from a predefined baseline by extensively modeling extensive normal operational data [10]. Early efforts primarily employed multivariate statistical analysis [11], monitoring key statistical indicators in data streams to detect anomalies. This was followed by the introduction of traditional machine learning techniques, such as One-Class Support Vector Machine (One-Class SVM) [12]. One-Class SVM utilizes a kernel function to map data into a high-dimensional space and construct a compact boundary enclosing normal samples. More recently, deep learning-based methods have emerged, including reconstruction-based methods using shallow Autoencoders [13,14] and boundary-based techniques like Deep SVDD [15]. Fundamentally, these methods seek to learn a unified model capable of accurately capturing the characteristics of normal behavior.

However, the fundamental limitation of both reconstruction-based and boundary-based detection methods lies in their reliance on overly idealized prior assumption. This core assumption is in sharp conflict with the intrinsic complexity of the IoV environment. IoV data is not only high-dimensional, dynamic, and non-linear, but more critically, its normal behavior is inherently multi-modal. Capturing such diverse behavioral patterns with a single reconstruction model or a unified geometric boundary is exceedingly challenging. This gives rise to a two dilemma. On one hand, to encompass all normal behavior patterns, the model must adopt a relaxed decision boundary. This risks accepting certain attack samples as normal, resulting in a high false negative rate. On the other hand, in striving for a tight boundary that rigidly excludes anomalies, the model inevitably misclassifies some borderline normal behaviors as attacks. This leads to a high false positive rate. Consequently, these methods are constrained by an intrinsic and difficult-to-resolve trade-off between detection accuracy and false alarm rates.

These limitations underscore a critical research gap, as existing methods frequently face a challenging trade-off between detection accuracy and false alarm rates. This issue stems from two fundamental factors: the poor quality of training data and the overly simplistic assumptions underlying detection models. To overcome these limitations, this paper introduces a novel zero-day attack detection framework for the IoV, named the Feature-Enhanced Inverse Mapping-based Zero-Day Detection method (IM-ZDD). To address the root cause of low-quality training data, we first design a feature enhancement generation module. Rather than relying on conventional data augmentation techniques, this module employs a Conditional Generative Adversarial Network (CGAN). This CGAN incorporates multiple generators and discriminators and is coupled with a novel ensemble dynamic focusing loss function. This architecture enables deep learning and refinement of the underlying distribution of the original normal data, with the goal of generating a large-scale, high-fidelity synthetic dataset that is both statistically robust and internally consistent. The resulting dataset exhibits significantly enhanced pattern coherence and sharper feature boundaries compared to the original data. Building on this high-quality dataset, we construct the Learned Inverse Mapping Detection (LIMD) module. The key innovation of this module lies in its exclusive reliance on the synthetic data for adversarial training. This allows it to construct a highly compact and precise feature space that comprehensively represents normal behaviors. This strategy effectively mitigates the influence of noise, redundancy, and pattern ambiguity in the original data. It thereby eliminates the dependence of traditional methods on simplified geometric or reconstruction-based assumptions and markedly improves detection sensitivity to unknown attacks. During the detection phase, each test sample undergoes the same feature enhancement mapping and normalization procedures as the training data to ensure distributional consistency. The processed sample is then input into the trained LIMD module. There, its anomaly score is computed based on the deviation of its inverse-mapped representation from the learned normal feature space. Extensive experiments on the F2MD IoV simulation platform demonstrate that IM-ZDD significantly outperforms state-of-the-art methods in terms of detection accuracy and false alarm rate. Furthermore, ablation studies validate the critical contribution of both core components, feature enhancement and inverse mapping detection, to the overall effectiveness of the proposed method.

The main contributions of this paper are as follows:

(1) We propose a novel feature enhancement generation framework to improve the quality of training data. Specifically, we designed a Conditional Generative Adversarial Network architecture composed of multiple generators and discriminators, complemented by a new ensemble dynamic focus loss. This design not only improves the stability of the generation process but also learns the underlying distribution of the original normal data and refines its core features, thereby generating a high-quality training set with more consistent patterns and sharper boundaries.
(2) We construct a Learned Inverse Mapping Detection module that breaks the reliance of traditional methods on simplified prior assumptions. Unlike conventional approaches that depend on reconstruction errors or geometric boundaries, this module adversarially learns a precise inverse mapping from high-dimensional data space to a low-dimensional latent space. When trained on the feature-enhanced synthetic dataset, the module captures a more compact and discriminative representation of normal behavior, significantly increasing the detection sensitivity for previously unseen attacks.
(3) We conduct comprehensive experimental validation on the F2MD IoV simulation platform and multiple public datasets. Comparative results demonstrate that the proposed IM-ZDD method substantially outperforms a range of state-of-the-art baseline models in terms of detection accuracy, robustness, and real-time performance. Moreover, extensive ablation studies confirm the effectiveness and superiority of each core component within the proposed framework.

## 2. Related Work

This section aims to provide a systematic review of the research progress in zero-day attack detection. It begins by examining the two predominant approaches to this problem: transfer learning-based detection and anomaly-based detection. For each approach, we explore its fundamental principles, representative methodologies, and inherent limitations. Building on this foundation, the review then narrows its focus to the specific application domain of the Internet of Vehicles (IoV). We analyze how these general-purpose methods have been applied and adapted in existing studies and conclude by summarizing the unique challenges they encounter within the distinctive context of the IoV environment.

### 2.1. Zero-Day Attack Detection Based on Transfer Learning

Given the pronounced scarcity of zero-day attack samples, an intuitive approach is to leverage transfer learning [16]. This involves transferring knowledge acquired from related or historical attacks to detect novel, sample-free threats. The core assumption underlying this approach is that different attacks share latent correlations within the feature space [17]. For example, the SALT framework [18] aligns the feature distributions of source and target domains in smart home scenarios through adversarial domain adaptation. To further enhance transfer efficiency, some studies have integrated unsupervised learning techniques to fuse complementary knowledge from different tasks [19]. However, successful transfer learning requires a sufficiently strong correlation between the source and target domains. When a significant domain gap exists, the efficacy of knowledge transfer diminishes drastically. This can potentially lead to negative transfer due to the interference of irrelevant knowledge, which adversely impacts detection performance [20].

To address this challenge, researchers have proposed mitigation strategies. These include methods like manifold alignment to reduce distributional disparities or direct fine-tuning using a limited number of known attack samples [8]. Nonetheless, these methods invariably depend on high-quality source data. To further alleviate the reliance on authentic attack samples, some research has explored the use of Generative Adversarial Networks (GANs). The goal is to synthesize novel attack data from a limited number of known samples, thereby augmenting the source domain [21]. However, existing studies [22] indicate that samples generated by standard GANs often only resemble real data in their superficial statistical features. They fail to replicate the complex internal logic and semantic patterns of authentic attacks. As a result, such synthetic data struggles to provide a meaningful information gain for knowledge transfer and thus offers limited benefit for improving model performance.

### 2.2. Zero-Day Attack Detection Based on Anomaly Detection

In light of the limitations of transfer learning, anomaly-based detection methods have gained widespread adoption due to their inherent adaptability to unknown patterns. These methods operate without reliance on any attack samples. Their core principle is to build a model of normal behavior on extensive normal data and then identify any data samples that this model cannot effectively represent as anomalies [3]. At its core, this approach reframes detection as a problem of verifying a system’s state against a learned model of normality, a concept theoretically grounded in state estimation and critical observability, as studies in domains such as discrete event systems [23]. Early research in this domain primarily employed statistical methods (e.g., multivariate analysis) [24] and traditional machine learning models (e.g., One-Class SVM) [12]. While these approaches perform adequately on low-dimensional and linearly separable datasets, their limited capacity to model complex data makes them insufficient for handling high-dimensional, non-linear scenarios commonly encountered in practice.

With the emergence of deep learning, reconstruction-based methods, represented by Autoencoders (AE) [13,14], have become a mainstream approach. Trained on a large volume of normal samples, these models learn to accurately compress and reconstruct normal patterns. When presented with an anomalous input, the model typically yields a high reconstruction error due to its unfamiliarity with the pattern. However, this approach hinges on the assumption that anomalies cannot be reconstructed well. In complex scenarios, where the diversity of normal behaviors must be accommodated, the model often develops an overly generalized reconstruction capability. As a result, it may inadvertently reconstruct subtle anomalies with high fidelity, leading to an inevitable increase in false negatives [25].

To address this shortcoming, boundary-based deep learning methods, such as Deep SVDD [14], have been applied to zero-day attack detection. These methods aim to map all normal data into a specific feature space using a deep neural network. They then enclose the data within a tight geometric volume, such as a minimum-volume hypersphere. However, the geometric assumption imposed by such models tend to be overly simplistic and idealistic, especially when applied to real-world data that often exhibit complex, multi-modal topological structures. This mismatch between the assumed and actual data distribution can cause a significant divergence between the learned decision boundary and the true structure of the data. This ultimately leads to elevated rates of both false positives and false negatives.

### 2.3. Zero-Day Attack Detection Methods in the IoV Domain

When the aforementioned general-purpose methods are applied to the highly dynamic and safety-critical domain of IoV, their inherent limitations become even more pronounced. The extreme complexity of the IoV environment and the pronounced multimodality of vehicle behavior (e.g., acceleration, crawling in congestion, and normal cruising) make it exceptionally difficult to distinguish between the wide variations in normal behavior and genuine attacks. As a result, directly applying traditional anomaly detection methods [26,27] often leads to high false positive rates due to their inability to adapt to such drastic pattern shifts. On the other hand, applying transfer learning [28,29] typically requires high-quality labeled attack data, which remains exceedingly scarce in IoV scenarios.

Recently, leveraging Conditional Generative Adversarial Networks (CGANs) for few-shot learning has emerged as a promising research direction. GANs can utilize a limited set of labeled samples to generate diverse and representative synthetic samples, thereby alleviating the problem of data scarcity [30]. In related domains such as Unmanned Aerial Vehicle (UAV) networks, prior studies have integrated CGANs with detection models to develop distributed and collaborative intrusion detection framework. These studies have demonstrated their effectiveness in sample-imbalanced scenarios [31].

Motivated by this line of research, this paper integrates a generative model with an anomaly detection method to realize a precise and efficient solution for zero-day attack detection in the IoV. First, a CGAN is employed to perform feature enhancement on the original normal data. This generates a large-scale synthetic dataset with well-defined feature boundaries. Then, under the anomaly detection paradigm, we design a novel learned inverse mapping module. This module is trained on the high-quality synthetic data to enable efficient and robust identification of zero-day attacks.

## 3. Method

This section presents the design philosophy and technical implementation of the IM-ZDD framework. A summary of the notations used throughout this paper can be found in Appendix A. We will explain how the feature enhancement generation and inverse mapping detection modules work collaboratively to address the challenges of sample scarcity and unknown patterns. We then delve into the detailed design of the feature enhancement generation module. which features an ensemble architecture comprising multiple generators and discriminators, along with the proposed Ensemble Dynamic Focus Loss that effectively refines the core features of the original data. Lastly, we provide a detailed analysis of the implementation of the Learned Inverse Mapping Detection module. This includes its network architecture, adversarial training mechanism, and the way it leverages high-quality synthetic data to construct a precise criterion for normality.

### 3.1. Overall Framework Architecture

**Figure 1:**
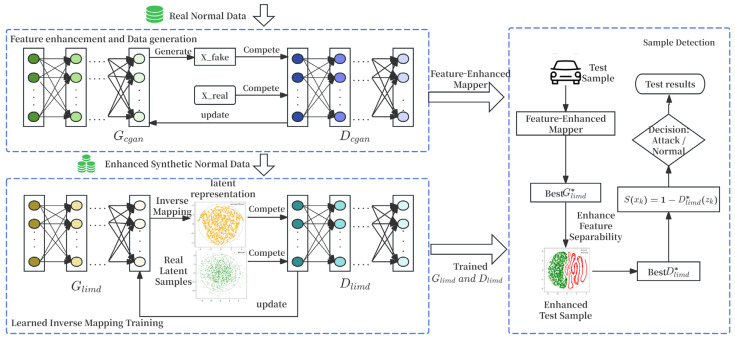
The two-stage architecture of the proposed IM-ZDD framework, detailing the feature enhancement and inverse mapping detection modules.

To address the performance limitations of traditional anomaly detection models caused by data scarcity in the IoV environment, this paper proposes a feature-enhanced inverse mapping framework for zero-day attack detection, termed IM-ZDD. As illustrated in Figure 1, the framework consists of two primary components: the Feature Enhancement Generation Module and the Learned Inverse Mapping Detection Module.

The Feature Enhancement Generation Module is designed to address the issue of low-quality training data. It adopts an ensemble Conditional GAN architecture. This architecture incorporates multiple parallel generators and discriminators to ensure both the stability of the generation process and the diversity of the synthesized samples. Within this module, we introduce the Ensemble Dynamic Focus Loss, which effectively amplifies the key feature differences between normal data and samples near the potential anomaly boundary during training. Utilizing only the original normal data, this module efficiently generates a large-scale synthetic normal dataset. This dataset is characterized by high pattern consistency and sharp feature boundaries, providing high-quality training input for the subsequent detection model.

The Learned Inverse Mapping Detection module is developed to overcome the excessive generalization problem common in traditional reconstruction-based methods. Rather than relying on reconstruction error, this module adopts an adversarial learning framework consisting of a Distribution Converter and a Latent Space Discriminator. The Distribution Converter learns a precise inverse mapping from the high-dimensional data space to a predefined low-dimensional latent space, while the Latent Space Discriminator assesses the fidelity of this mapping within the latent space. To guide the model toward learning a more compact and accurate representation of normal behavior than conventional methods, the training process integrates a dynamic focusing loss mechanism.

During the detection phase, a test sample first passes through the feature enhancement mapping, projecting it from the original feature space into the enhanced feature space. The enhanced sample is then normalized using the same procedure applied during training to ensure distribution alignment, and subsequently fed into the trained LIMD module. Within the module, the Distribution Converter maps the input sample to the predefined latent space. The Latent Space Discriminator then evaluates how closely this latent representation aligns with the learned normal distribution. A normal sample, being consistent with the training data patterns, is mapped into the core region of the normal distribution, resulting in an anomaly score near 0. In contrast, an anomalous sample, due to its unfamiliar pattern, cannot be effectively mapped. It exhibits significant deviation in the latent space, ultimately yielding a high anomaly score close to 1.

### 3.2. Feature Enhancement Generation Module

To enable effective feature enhancement of IoV data, this module is constructed using a Conditional Generative Adversarial Network (CGAN). Traditional CGANs typically adopt a single generator-discriminator architecture. However, they are prone to mode collapse and produce samples with insufficient diversity when dealing with complex, multi-modal distributions like those in IoV data. To address this limitation, we extend the traditional architecture into an ensemble structure composed of $N_{G}$ generators ${\left\{G_{i}\right\}}_{i=1}^{N_{G}}$ and $N_{D}$ discriminators ${\left\{D_{j}\right\}}_{j=1}^{N_{D}}$. To effectively train this ensemble architecture, we propose the Ensemble Dynamic Focus Loss (EDFL), designed to deeply learn and refine the intrinsic features of the original normal data.

The core objective of this module is to mitigate pattern ambiguity and noise inherent in the original normal data. Directly training a model on such data often yields overly generalized decision boundaries, thereby increasing the false positive rate. To address this issue, the proposed ensemble architecture employs multiple generators to enhance the diversity and coverage of the synthetic data, while collaborative learning among multiple discriminators stabilizes the training process. The EDFL further incorporates an adaptive weighting mechanism that emphasizes samples located near the decision boundary between normal and potentially anomalous behavior. Its internal dynamic modulation factor reduces the weight of easily classified samples in the loss calculation, compelling the generators to synthetic data with sharper and more well-defined feature boundaries. EDFL is built upon the standard Binary Cross-Entropy (BCE) loss, whose general form is expressed in Equation (1):

(1) $$L_{BCE}\left(p,y\right)=-\left[y\cdot \ln\left(p\right)+\left(1-y\right)\cdot \ln\left(1-p\right)\right]$$

where $y\in \left\{0,1\right\}$ is the ground-truth label, and $p\in \left[0,1\right]$ represents the model’s predicted probability of the label being 1.

The core innovation of EDFL lies in its adaptive weighting mechanism, which leverages historical discriminator confidence to foster collaborative learning among the ensemble of discriminators. For the *j*-th discriminator $D_{j}$, the adversarial loss component for a real data sample x is weighted by a dynamic modulation factor. This factor is computed based on the discriminator’s average confidence on real data from previous batches, denoted $p_{j,hist}$, as shown in Equation (2):

(2) $$w_{j}^{\left(k\right)}={\left(1-p_{j,\mathrm{hist}}\right)}^{\gamma }$$

Here, γ is a focusing parameter that controls the strength of modulation.

The intuition is that if a discriminator has historically performed well, its weight for easily classified samples in the current batch is reduced. This enables the model to focus its learning on hard-to-classify samples near the decision boundary. The final weighted loss for real data, $L_{real,j}$, is then formulated as shown in Equation (3):

(3) $$L_{real,j}=\frac{1}{m}\sum_{k=1}^{m}w_{j}^{\left(k\right)}\cdot \left[L_{BCE}\left(D_{j}\left(x^{\left(k\right)}\right),1\right)+L_{BCE}\left(D_{j}\left(x^{\left(k\right)}\right),y^{\left(k\right)}\right)\right]$$

Similarly, the loss for fake data, $L_{fake,j}$, adopts the same modulation mechanism, as shown in Equation (4):

(4) $$L_{fake,j}=\frac{1}{n}\sum_{k=1}^{n}w_{j}^{\left(k\right)}\cdot \left[L_{BCE}\left(D_{j}\left(x_{fake}^{\left(k\right)}\right),0\right)+L_{BCE}\left(D_{j}\left(x_{fake}^{\left(k\right)}\right),y_{fake}^{\left(k\right)}\right)\right]$$

For the *j*-th discriminator, its total loss $L_{D_{j}}$ is computed as the weighted sum of its losses on real and fake data, formulated in Equation (5). For the *i*-th generator, $G_{i}$, the objective is to minimize a loss function $L_{G_{i}}$ that aims to fool all $N_{D}$ discriminators. This generator loss is defined in Equation (6):

(5) $$L_{D_{j}}=\alpha L_{real,j}+\left(1-\alpha \right)L_{fake,j}$$

(6) $$L_{G_{i}}=\frac{1}{N_{D}}\sum_{j=1}^{N_{D}}\frac{1}{n}\sum_{k=1}^{n}w_{i,j}^{\left(k\right)}\cdot \left[L_{BCE}\left(D_{j}\left(G_{i}\left(z^{\left(k\right)},y^{\left(k\right)}\right)\right),1\right)+L_{BCE}\left(D_{j}\left(G_{i}\left(z^{\left(k\right)},y^{\left(k\right)}\right)\right),y^{\left(k\right)}\right)\right]$$

In Equation (6), the modulation factor $w_{i,j}^{\left(k\right)}$ is dynamically adjusted based on the historical success rate of generator $G_{i}$ in fooling discriminator $D_{j}$. This correlation incentivizes the generator to continuously improve, ultimately achieving the enhancement of key data features through the adversarial game.

Upon completion of the training process described above, a set of well-trained generators ${\left\{G_{i}^{*}\right\}}_{i=1}^{N_{G}}$ is obtained. These generators are capable of producing a feature-enhanced synthetic dataset. To ensure compatibility with the input requirements of the subsequent detection module, normalization is performed as outlined in Algorithm 1.
**Algorithm 1** Feature Space Normalization and Adaptation**Input:** A feature-augmented synthetic dataset $D_{in}=\left\{x^{\left(1\right)},\ldots ,x^{\left(N\right)}\right\}$, where $x^{\left(k\right)}\in {\mathbb{R}}^{d}$**Output:** A normalized dataset $D_{out}$ and the normalization transformation T1:   Initialize two vectors: $v_{\min}\in {\mathbb{R}}^{d}$ $v_{\max}\in {\mathbb{R}}^{d}$2:   **for** j = 1 to d **do**3:   Calculate the minimum value of the j-th feature: $v_{\min,j}\leftarrow \min_{k=1}^{N}\left(x_{j}^{\left(k\right)}\right)$4:   Calculate the maximum value of the j-th feature: $v_{\max,j}\leftarrow \max_{k=1}^{N}\left(x_{j}^{\left(k\right)}\right)$5:   **end for**6:   Calculate the dynamic range vector of the feature space: $r\leftarrow v_{\max}-v_{\min}$7:   Define the Min-Max scaling transformation T, where for any input vector x:•   T(x) is the transformation function such that ${T(x)=(x-v}_{\min})/r$, where the division is element-wise.8:   Initialize an empty dataset for the output: $D_{out}\leftarrow \emptyset$9:   for each sample $x^{\left(k\right)}\in D_{in}$ **do**10:  Apply the scaling transformation to normalize the sample: $x\prime^{\left(k\right)}\leftarrow T\left(x^{\left(k\right)}\right)$11:  Add the normalized samples to the output dataset: $D_{out}\leftarrow D_{out}\cup \left\{x\prime^{\left(k\right)}\right\}$12:  end for13:  return $D_{out},T$

The normalization process begins by traversing the dataset to identify the minimum and maximum values for each feature dimension. Then, Min-Max Scaling is applied to linearly map all data points into the [0, 1] interval. This normalization eliminates numerical scale differences between features, thereby improving the training stability and convergence speed of the subsequent detection model.

### 3.3. Learned Inverse Mapping Detection Module

To overcome the reliance of traditional anomaly detection methods on reconstruction error or simplified geometric assumptions, this paper designs a Learned Inverse Mapping Detection module. The core idea is inspired by the inverse application of Generative Adversarial Networks. While a standard GAN learns a forward mapping from a low-dimensional latent space to a high-dimensional data space through adversarial training, this work reverses the process in light of the high complexity of IoV data. Specifically, the adversarial learning paradigm is employed to learn an inverse mapping function. This function accurately projects high-dimensional normal data into a predefined, compact low-dimensional latent space. The LIMD module consists of two main components: a Distribution Converter, $G_{limd}$, and a Latent Space Discriminator, $D_{limd}$.

This architecture decomposes the traditional single-autoencoder detection paradigm into two independent components with well-defined functionalities. The Distribution Converter $G_{limd}$ is responsible for learning an accurate inverse mapping from the original high-dimensional data space to a predefined low-dimensional latent space. Operating within this structured latent space, the Latent Space Discriminator $D_{limd}$ evaluates whether the latent representation of a sample, as produced by $G_{limd}$, conforms to the target distribution. The LIMD module is trained exclusively on the high-quality synthetic normal data generated in the previous stage. This enables it to establish a highly precise and compact normality baseline. This decoupling of the model architecture, combined with the use of high-quality training data, enables LIMD to construct a normal behavior distribution that is significantly tighter and has clearer boundaries than traditional methods. This substantially enhances the detection sensitivity for unknown attacks.

**Figure 2:**
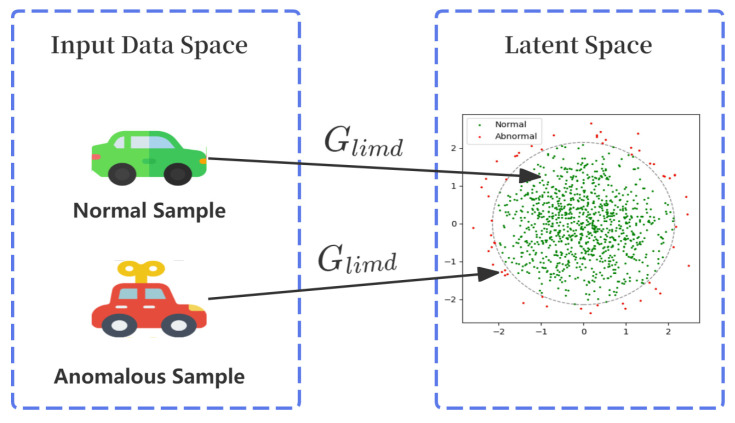
Visualization of the detection principle: Normal samples are mapped inside the target latent distribution yielding low anomaly scores, while anomalous samples are mapped outside, yielding high scores.

To conceptually illustrate this detection mechanism, Figure 2 shows its behavior on both normal and anomalous samples. A normal sample, consistent with the distribution learned during training, is mapped by $G_{limd}$ into the core region of the target latent distribution. As a result, $D_{limd}$ assigns it a high confidence score (close to 1), yielding a low anomaly score. In contrast, an anomalous sample cannot be effectively mapped. Its latent representation deviates from the normal distribution, leading a low confidence score (close to 0) and consequently a high anomaly score.

The training of the LIMD module follows a standard adversarial learning framework. For the training data, which is the feature-enhanced synthetic normal dataset, we sample a dataset $D_{z}$ containing $N_{z}$ real latent samples from a target latent distribution $p_{z}$. The optimization objective for the discriminator $D_{limd}$ is to minimize its classification error—that is, to accurately distinguish between the real latent samples from the target distribution $p_{z}$ and the fake latent samples generated by the converter $G_{limd}$. Its loss function, $L_{D_{limd}}$, for a single batch is defined in Equation (7):

(7) $$L_{D_{limd}}=-\left[\frac{1}{n_{z}}\sum_{i=1}^{n_{z}}\ln\left(D_{limd}\left(z^{\left(i\right)}\right)\right)+\frac{1}{m}\sum_{k=1}^{m}\ln\left(1-D_{limd}\left(G_{limd}\left(x^{\left(k\right)}\right)\right)\right)\right]$$

where $n_{z}$ and m are the batch sizes for $D_{z}$ and $D_{normal}$ respectively.

The optimization objective for the Distribution Converter $G_{limd}$ is to generate latent samples that can fool the discriminator $D_{limd}$, making it believe that these converted samples also originate from the target distribution $p_{z}$. Its loss function, $L_{G_{limd}}$, is defined as shown in Equation (8):

(8) $$L_{G_{limd}}=-\frac{1}{m}\sum_{k=1}^{m}\ln\left(D_{limd}\left(G_{limd}\left(x^{\left(k\right)}\right)\right)\right)$$

By alternately optimizing the loss functions $L_{D_{limd}}$ and $L_{G_{limd}}$ via gradient descent, the trained Distribution Converter $G_{limd}^{*}$ learns to precisely map all feature-enhanced normal data into the high-density regions of the target distribution $p_{z}$. Simultaneously, the discriminator $D_{limd}^{*}$ learns how to accurately recognize these regions.

Once training is complete, the IM-ZDD framework is ready to perform detection on unseen samples. Each test sample is first passed through the same feature enhancement mapping and normalization procedure used during training to ensure distributional alignment. In the detection phase, for any pre-processed test sample $x_{test}$, its anomaly score, $S\left(x_{test}\right)$, is directly determined by the output of the Latent Space Discriminator, as shown in Equation (9):

(9) $$S\left(x_{test}\right)=1-D_{limd}^{*}\left(G_{limd}^{*}\left(x_{test}\right)\right)$$

A normal sample, being consistent with the training data in its pattern, can be accurately mapped by $G_{limd}^{*}$ into the core region of the target distribution. As a result, its latent representation receive a high confidence score approaching 1 from $D_{limd}^{*}$, resulting in a final anomaly score close to 0. Conversely, an anomalous sample, whose pattern is unknown, cannot be effectively mapped. Its representation in the latent space will exhibit a significant deviation from the normal distribution, receiving a low confidence score near 0 and thus yielding a high anomaly score close to 1. Through this scoring mechanism based on latent space distribution matching, LIMD breaks free from the reliance on reconstruction error or simplified geometric assumptions. It provides greater robustness and adaptability when dealing with the high-dimensional, dynamic characteristics of IoV data.

## 4. Experiments and Analyses

This section presents a comprehensive evaluation of the proposed feature-enhanced inverse mapping detection method (IM-ZDD). We evaluate its performance, robustness, and component effectiveness. First, we detail the experimental setup. This includes the characteristics and anomaly definitions of the datasets used, the selected evaluation metrics, and the baseline methods for comparison. Next, we conduct a thorough performance comparison between IM-ZDD and the baseline methods across different datasets and attack scenarios. This is followed by ablation studies to investigate the contributions of the core innovative components, namely the feature enhancement generation and the learned inverse mapping detection modules. Lastly, we examine the model’s sensitivity to key hyperparameters and assess its detection latency to validate the overall effectiveness and practical application potential of IM-ZDD.

### 4.1. Experimental Setup

Our research is primarily evaluated on feature data generated by the F2MD [32] (Framework for Misbehavior Detection in Vehicular Networks) simulation platform. This platform is capable of modeling complex IoV environments containing both normal traffic and various types of attacks. In our experiments, we focus on four representative attack types from the F2MD dataset: Denial of Service (DoS), Random Speed (RS), Random Position Offset (RPO), and Disruptive attacks. Additionally, we construct a Multi-attack scenario that incorporates all of the above attack types. To further assess the generality of our framework, we incorporate four widely used public anomaly detection datasets: Thyroid Disease [33], bank-additional-full [34], celeba_baldvnonbald [35], and kdd99-unsupervised [36]. For all datasets, samples are categorized as either normal or anomalous according to their standard definitions. During training, the feature enhancement generation module is exclusively trained on normal samples from the original dataset. The resulting synthetic normal dataset then serves as the sole training source for the Learned Inverse Mapping Detection module.

To comprehensively evaluate model performance, we employ multiple metrics. The Area Under the ROC Curve (AUC) is used to assess the model’s overall ranking ability and discriminative performance. The F1-Score, as the harmonic mean of Precision and Recall, serves as a key indicator for classification accuracy on imbalanced datasets. The False Positive Rate (FPR) is used to assess the degree to which a model interferes with normal behavior in practical applications.

To objectively evaluate the performance of the IM-ZDD framework, we compare it against six representative state-of-the-art anomaly detection methods: DAGMM [37], ICL [15], PreNet [38], SLAD [39], FGAN [40], and FSCGAN [41]. All experiments were conducted under identical hardware conditions and data partitioning settings as those used for IM-ZDD. Additionally, the hyperparameters for each baseline model were carefully fine-tuned to ensure a fair and reliable comparison.

We tuned the key hyperparameters of all baseline methods to ensure a fair comparison. The selected settings were primarily guided by the recommendations provided in their original papers. Table 1 presents the hyperparameter search spaces along with the final values adopted in our experiments.

For dataset partitioning, normal samples from each dataset were divided such that 70% were allocated for the training phase and the remaining 30% were retained as the normal portion of the test set. The test set additionally incorporated all available anomaly samples. The feature enhancement module was trained for 200 epochs and the LIMD module for 100 epochs, with a batch size of 32. All networks were optimized using Adam with a learning rate of 1 × 10^−4^. Key hyperparameters were configured as follows: the focusing parameter γ in the EDFL was set to 2.0, and the latent space dimension of the LIMD module was fixed at 4. All experiments were implemented in Python (version 3.12.0) using the PyTorch (version 2.3.1) framework and executed on an NVIDIA GeForce RTX 3060 GPU.

### 4.2. Performance Comparison and Analysis

**Figure 3:**
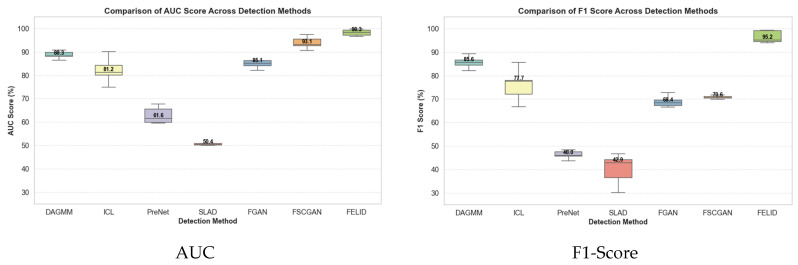
Comparative performance of IM-ZDD against baseline methods on the F2MD dataset, evaluated by AUC and F1-Score distributions across all attack scenarios.

This section comparatively analyzes the performance of the IM-ZDD framework against six baseline methods on the F2MD IoV dataset. Table 2 and Table 3 report the AUC and F1-Score values for each model across different attack scenarios. As shown in Table 2, IM-ZDD achieves an average AUC of 98.25% across all attack types, outperforming the next-best method, FSCGAN (93.85%), by 4.4 percentage points. Particularly in the DoS and Multi-attack scenarios, IM-ZDD achieves AUC scores of 99.37% and 99.73%, respectively. These results indicate that the normality criterion constructed by IM-ZDD is highly precise and compact. This enables effective discrimination between normal and anomalous distributions and demonstrates superior overall ranking performance. From the F1-Score results in Table 3, IM-ZDD achieves an average F1-Score of 96.41%. This exceeds the runner-up, DAGMM (85.61%), by more than 10 percentage points and further confirms the strength of IM-ZDD in accurately identifying diverse and complex attack patterns within the IoV environment. Collectively, these results demonstrate that IM-ZDD successfully addresses the limitations of traditional methods in modeling original data distributions, delivering outstanding detection effectiveness. A visual summary of these comparative results is provided in Figure 3, offering an intuitive understanding of performance differences across various attack scenarios.

Regarding the risk of overfitting, our two-stage approach provides inherent mitigation. Instead of memorizing specific training instances, the framework learns a comprehensive model of normal behavior. In the initial feature enhancement stage, a large-scale, high-quality synthetic dataset is generated from the limited original samples. This dataset is statistically more robust and diverse, thereby reducing the likelihood that the subsequent LIMD module overfits to noise or incidental patterns in the original data. The consistently strong performance across all test scenarios further demonstrates that the model captures a generalizable representation of normality.

To further validate the generalizability of the core design philosophy underlying IM-ZDD, we evaluated its performance on several public benchmark datasets. As shown in Table 4, IM-ZDD consistently demonstrates excellent detection performance across these diverse datasets. For example, on the bank-additional-full dataset, it achieves an AUC of 99.96% and an F1-Score of 99.75%. On the kdd99-unsupervised dataset, the AUC and F1-Score reached 98.86% and 99.87%, respectively. These cross-domain experimental results fully validate the generalizability of our core methodology. By first enhancing data quality before conducting precise modeling, our framework provides a feasible and effective technical pathway for other detection tasks that face similar challenges of data scarcity and complex distributions.

**Figure 4:**
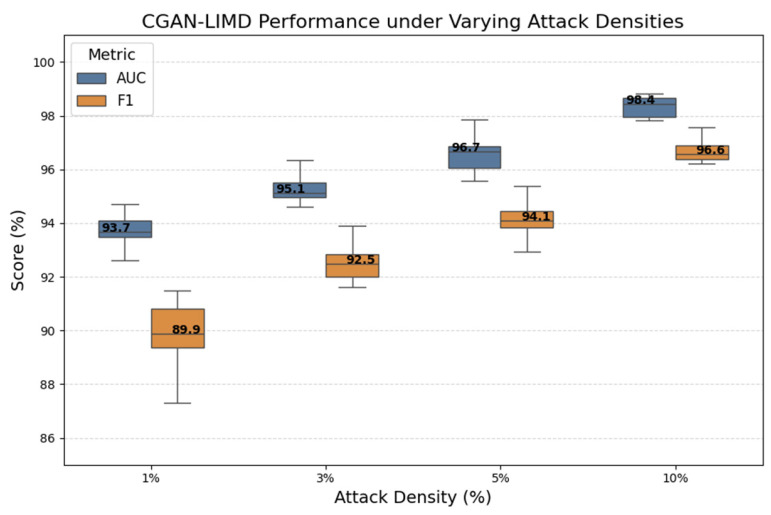
Performance stability of IM-ZDD evaluated under varying attack densities (1% to 10%) in the Multi-attack scenario.

To evaluate the performance stability of the IM-ZDD framework under varying attack intensities, we conducted an experiment on the impact of changing attack densities on model performance. This experiment was conducted in the Multi-attack scenario, where the proportion of attack samples in the test set was set to four levels: 1%, 3%, 5%, and 10%. This range simulates a spectrum of attack conditions from sporadic to frequent occurrences. Figure 4 presents the corresponding AUC and F1-scores of IM-ZDD under these different attack densities. The results reveal a positive correlation between model performance and the attack density in the test set. However, even in the sparse scenario where the attack density was limited to just 1%, the median AUC and F1 scores remained high at approximately 94% and 90%, respectively. No significant performance degradation was observed. These findings underscore the exceptional robustness of the IM-ZDD framework in identifying zero-day attacks, even when they occur infrequently and stealthily. This robustness further highlights the practical applicability of IM-ZDD in real-world IoV environments, where attack instances may be both rare and highly unpredictable.

### 4.3. Ablation Experiments

To quantitatively evaluate the necessity and effectiveness of each key innovative component within the IM-ZDD framework, we conducted a series of ablation studies. These experiments were designed to evaluate: (1) the overall contribution of the feature enhancement generation module, (2) the impact of the Ensemble Dynamic Focus Loss within this module, and (3) the performance advantages of the Learned Inverse Mapping Detection (LIMD) module over traditional detection paradigms. The results are summarized in Table 5.

First, to examine the overall contribution of the feature enhancement generation module, we constructed a baseline variant named LIMD-Raw. In this variant, the generation module is completely removed, and the LIMD module is trained directly on the limited, original normal data. The results reveal that LIMD-Raw achieves an average F1-Score of only 80.23%, exhibiting a significant performance drop compared to the complete IM-ZDD framework. This underscores the critical role of the feature enhancement module in producing a large-scale, high-quality synthetic normal dataset with sharp feature boundaries. This dataset provides a strong data foundation for the LIMD module to learn a precise model of normal behavior.

Second, to assess the effectiveness of the Ensemble Dynamic Focus Loss in the generation module, we constructed a CGAN (Basic Loss) + LIMD variant. This variant removes the dynamic weighting mechanism based on historical performance during the generator’s training, retaining only the basic adversarial loss. The experimental results show that this variant’s average F1-Score drops to 84.27%. This validates the significant role of the dynamic weighting mechanism in improving the quality of the generated data. By guiding the model to focus on hard-to-classify samples, this mechanism effectively enhances the expressive power for boundary samples, thereby boosting the quality of the generated data.

Finally, to evaluate the advantages of the LIMD module compared to traditional detection methods, we constructed an IM-ZDD (AE-Rule) variant. This variant replaces the LIMD module with a standard autoencoder-based detection method that relies on reconstruction error. It is trained on the same high-quality normal data generated by our feature enhancement module. The results show that this variant’s F1-Score plummets to 54.54%. This highlights the limitations of traditional autoencoder-based detection in handling high-dimensional, complex IoV data. This result clearly demonstrates that the proposed learned inverse mapping module holds significant advantages in both anomaly modeling and recognition.

To quantify the contribution of the Ensemble Dynamic Focus Loss, we conducted additional comparative experiments. In these tests, the loss function within the feature enhancement module was varied, while the rest of the IM-ZDD framework remained unchanged. This allowed us to assess the direct impact of different loss designs on final detection performance.

**Figure 5:**
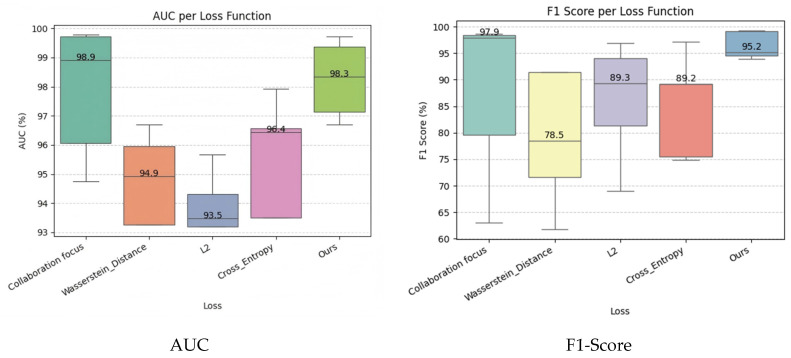
Performance comparison of IM-ZDD using different loss functions for the feature enhancement module.

The results are presented in Table 6 and Figure 5. With our proposed Ensemble Dynamic Focus Loss (Ours), the IM-ZDD framework consistently achieves superior performance. For instance, it reaches an F1-Score of 99.23% in the Multi-attack scenario. Figure 5 also shows that our loss function yields not only the highest mean performance but also the most concentrated distribution, indicating stronger stability. In contrast, alternative losses like Wasserstein distance or L2 loss result in significantly lower performance and larger fluctuations.

These findings demonstrate that the Ensemble Dynamic Focus Loss is key to high-quality feature enhancement. Feature enhancement aims to refine and amplify ambiguous patterns from the original data, making them more discriminable in synthetic samples. Our proposed loss function uses a performance-adaptive weighting mechanism to guide the CGAN. Instead of merely mimicking superficial data, it reconstructs the essence of normal behavior. This mechanism improves the quality of the generated samples and reduces the learning difficulty for the LIMD module. Consequently, LIMD can construct a more precise and robust decision boundary, which underpins the performance of the entire framework.

### 4.4. Detection Latency Analysis

**Figure 6:**
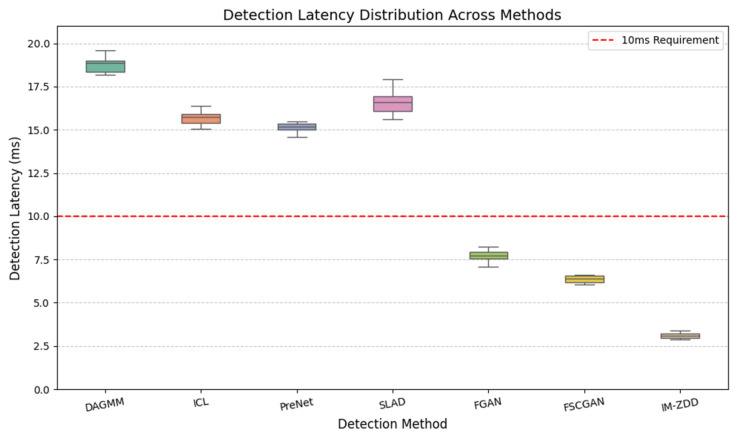
Per-sample detection latency comparison, showing IM-ZDD’s efficiency against baseline methods and the 10 ms real-time requirement.

In addition to detection accuracy, computational efficiency and detection latency are crucial indicators for determining whether a detection method can be deployed in environments with stringent real-time requirements, such as the Internet of Vehicles. This section quantitatively analyzes the latency performance of the IM-ZDD method at the detection phase and compares it with two high-performing baselines, DAGMM and SLAD. Figure 6 illustrates the detection latency of each method when processing a single data sample. The experimental data reveals that the median detection latency of IM-ZDD is approximately 3 ms. This is significantly lower than the 18.5 ms of DAGMM and 16.5 ms of SLAD, demonstrating a strong computational advantage. The low latency of IM-ZDD is primarily attributed to the highly efficient architecture of its Learned Inverse Mapping Detection module. At the detection stage, once a test sample has been processed by the feature enhancement mapping, the system only needs to perform a single, non-iterative forward pass. First, the Distribution Converter $G_{limd}^{*}$ maps the sample to the latent space. Subsequently, the Latent Space Discriminator $D_{limd}^{*}$ performs a rapid evaluation of the resulting representation. This approach effectively circumvents the complex reconstruction process common in autoencoder-based methods, thereby significantly reducing computational overhead.

Industry standards typically require end-to-end latency for safety-critical vehicular communication to be under 10 ms. In our experiments, IM-ZDD consistently meets this real-time requirement. Considering both its detection performance and latency, IM-ZDD offers significant advantages in accuracy and robustness. Furthermore, its outstanding low-latency characteristic establishes it as an efficient and practical technical solution capable of meeting the stringent real-time demands of IoV.

## 5. Discussion

The core contribution of this paper lies in validating the effectiveness of the proposed IM-ZDD framework for zero-day attack detection in the Internet of Vehicles. Experimental results demonstrate that, compared to existing baseline methods, the proposed framework holds significant advantages across key metrics including detection accuracy, false positive rate, and detection latency. At the data level, to address the challenges of inconsistent quality and ill-defined feature boundaries in the original normal data, we designed the feature enhancement generation module. This module produces a large-scale, high-quality synthetic normal dataset with sharp boundaries. This dataset significantly improves the quality and expressiveness of the training data and lays a solid foundation for subsequent precise modeling. At the model level, we developed the Learned Inverse Mapping Detection module to overcome the limited discriminative power of traditional models based on simplified assumptions. This module learns a precise inverse mapping from a high-dimensional observation space to a low-dimensional latent space, establishing a more precise and robust normality criterion. This approach effectively captures the intrinsic structure and regularities of the feature data stream. It simultaneously reduces the false negative rate while significantly alleviating the challenge of high false positives. Nonetheless, this work has certain limitations. The core philosophy of our framework is to identify any deviation from a precisely learned model of normality. Consequently, the framework’s performance may be challenged when it confronts concept drift—the evolution of normal behavior patterns over time. Specifically, when new normal behavioral patterns that deviate from the original training distribution emerge in the IoV environment, the current model may misclassify them as anomalies. This could lead to an increase in the false positive rate. Therefore, a promising direction for future research is to integrate incremental learning mechanisms, such as online learning or continual learning, into the IM-ZDD framework. By introducing such mechanisms, the model could leverage new incoming data to dynamically and efficiently update its model of normal behavior. This would enable the framework to continuously adapt to evolving network environments, thereby significantly enhancing the model’s robustness in long-term deployments. In terms of practical deployment, IM-ZDD is designed to address both scalability and regulatory requirements. For scalability, its detection phase is highly efficient, requiring only a single, non-iterative forward pass with a median latency of 3 ms. This low computational overhead makes the framework suitable for deployment on both resource-constrained in-vehicle edge devices as well as high-throughput cloud platforms. Regarding regulatory compliance, automotive cybersecurity standards such as ISO/SAE 21434 [42] emphasize proactive risk management against unknown threats. By operating independently of attack signatures, IM-ZDD aligns with these standards, offering a mechanism to identify novel vulnerabilities throughout the vehicle lifecycle.

## 6. Conclusions

This paper addresses the critical challenge of zero-day attack detection in the Internet of Vehicles by introducing a novel feature-enhanced inverse mapping framework, IM-ZDD. The framework decouples the task into two distinct yet complementary stages: data quality optimization and detection model construction, effectively integrating high-quality data generation with high-sensitivity anomaly detection. In the data generation stage, IM-ZDD employs a multi-generator, multi-discriminator CGAN architecture coupled with the proposed Ensemble Dynamic Focus Loss. This configuration enables the synthesis of a large-scale, high-quality training set with sharp feature boundaries from limited original data. In the detection modeling stage, this generated dataset is used to train the Learned Inverse Mapping Detection module, which constructs a compact and precise normality distribution. This approach eliminates reliance on reconstruction errors and simplistic geometric assumptions and facilitates sensitive identification of previously unseen attack patterns. Comprehensive experimental evaluations confirm the effectiveness of the proposed framework. Across multiple attack scenarios, IM-ZDD consistently outperforms state-of-the-art baselines in terms of detection accuracy and robustness. Moreover, its efficient, feed-forward detection process satisfies the real-time constraints of IoV applications. Ablation studies further underscore the contribution of each core component, highlighting data optimization as a decisive factor in achieving superior detection performance.

Looking ahead, future work will explore several promising directions. First, we aim to incorporate diffusion-based generative techniques to further enhance feature representation quality. Second, we will investigate semi-supervised and self-supervised learning strategies to minimize dependency on initial labeled data. Lastly, we plan to extend the framework to support online learning, enabling it to adapt to evolving attack behaviors and concept drift in dynamic IoV environments.

## Figures and Tables

**Table 1:** Hyperparameter settings for the baseline methods.

Method	Search Space (Main Hyperparameters)	Final Value
DAGMM	Learning rate {1 × 10^−3^, 1 × 10^−4^}; Compression dim {8, 16, 32}; Gaussian components {2, 4, 8}	1 × 10^−4^/16/4
ICL	Learning rate {1 × 10^−3^, 1 × 10^−4^}; Contrastive temperature {0.1, 0.2, 0.5}	1 × 10^−4^/0.2
PreNet	Learning rate {1 × 10^−3^, 1 × 10^−4^}; Prior strength {0.1, 1, 10}	1 × 10^−4^/1
SLAD	Learning rate {1 × 10^−3^, 1 × 10^−4^}; Sliding window {32, 64, 128}	1 × 10^−4^/64
FGAN	Learning rate {1 × 10^−3^, 1 × 10^−4^}; Discriminator steps {1, 2}	1 × 10^−4^/1
FSCGAN	Learning rate {1 × 10^−3^, 1 × 10^−4^}; Class-balance λ {0.1, 0.5, 1}	1 × 10^−4^/0.5

**Table 2:** Comparative experiments on AUC values between IM-ZDD and the other 6 methods (The best ones are in Bold, and the second ones are underlined).

Attack Types	Detection Methods						
	DAGMM	ICL	PreNet	SLAD	FGAN	FSCGAN	IM-ZDD
DoS	89.82%	80.17%	61.60%	50.40%	82.15%	93.12%	**99.37%**
Disruptive	88.07%	90.05%	59.60%	50.81%	86.35%	92.68%	**96.70%**
RandomSpeed	90.82%	81.25%	59.85%	50.23%	86.24%	90.63%	**98.34%**
RandomPosOffset	88.34%	84.21%	65.61%	50.85%	85.12%	95.42%	**97.13%**
Multi-attack	86.51%	74.98%	67.75%	50.00%	84.17%	97.44%	**99.73%**
Average	88.71%	82.13%	62.88%	50.46%	84.80%	93.85%	**98.25%**
Rank	3.00	4.60	6.00	7.00	4.20	2.20	**1.00**

**Table 3:** Comparative experiments on F1-Score between IM-ZDD and the other 6 methods (The best ones are in Bold, and the second ones are underlined).

Attack Types	Detection Methods						
	DAGMM	ICL	PreNet	SLAD	FGAN	FSCGAN	IM-ZDD
DoS	85.61%	77.85%	47.47%	42.86%	66.60%	71.07%	**99.17%**
Disruptive	84.37%	85.59%	45.96%	44.19%	72.80%	71.68%	**94.53%**
RandomSpeed	89.31%	72.09%	43.67%	36.48%	69.60%	70.63%	**93.98%**
RandomPosOffset	86.67%	77.66%	45.64%	46.62%	67.20%	69.93%	**95.15%**
Multi-attack	82.09%	66.67%	48.40%	30.12%	68.38%	70.47%	**99.23%**
Average	85.61%	75.97%	46.23%	40.05%	68.92%	70.76%	**96.41%**
Rank	2.00	3.40	6.20	6.80	4.60	4.00	**1.00**

**Table 4:** Comparative Experiments on Multiple Datasets.

Dataset	Evaluation Indicators						
	AUC	F1	G-Mean	K-S	MCC	FPR	AUPRC
Multi-attack	99.73%	99.23%	99.13%	98.26%	98.26%	1.22%	99.13%
Thyroid	92.98%	99.26%	91.34%	83.10%	83.49%	13.93%	97.53%
bank-additional-full	99.96%	99.75%	99.98%	99.96%	99.96%	0.04%	99.69%
celebA_baldvsnonbald	99.90%	99.94%	97.08%	94.24%	94.40%	2.12%	99.93%
kdd99-unsupervised	99.86%	99.87%	99.87%	99.74%	99.72%	0.26%	99.15%

**Table 5:** Ablation experiments between IM-ZDD and IM-ZDD variants (The best ones are in Bold).

Comparison Models	Attack Types					Avg F1
	DoS	Disruptive	RandomSpeed	RandomPosOffset	Multi-Attack	
LIMD-Raw	94.51	85.67	60.31	78.34	82.31	80.23
CGAN (Basic Loss) + LIMD (Full)	96.28	89.04	63.76	84.39	87.87	84.27
CGAN + LIMD (AE-Rule)	61.28	54.97	50.04	48.16	58.27	54.54
IM-ZDD (Full)	**99.17**	**94.53**	**93.98**	**95.15**	**99.23**	**96.41**

**Table 6:** The detection performance of IM-ZDD under different loss function definitions (The best ones are in Bold).

Attack Types	DoS		Disruptive		RandomSpeed		RandomPosOffset		Multi-Attack	
Loss Function	AUC	F1	AUC	F1	AUC	F1	AUC	F1	AUC	F1
Collaboration focus	**99.78%**	98.66%	**98.92%**	**97.87%**	94.75%	63.07%	96.06%	79.63%	99.72%	98.37%
Wasserstein Distance	96.69%	78.48%	95.96%	91.48%	87.93%	71.64%	93.26%	61.82%	94.93%	91.43%
L2	93.48%	96.91%	95.68%	89.33%	89.69%	81.34%	94.32%	69.06%	93.20%	94.08%
Cross_Entropy	97.93%	89.19%	96.43%	89.19%	96.56%	74.81%	93.49%	75.52%	83.35%	97.17%
Ours	99.37%	**99.17%**	96.70%	94.53%	**98.34%**	**93.98%**	**97.13%**	**95.15%**	**99.73%**	**99.23%**

## Data Availability

The raw data supporting the conclusions of this article will be made available by the corresponding author upon reasonable request.

## Abbreviations

The following abbreviations are used in this manuscript:

IoV	Internet of Vehicles
CGAN	Conditional Generative Adversarial Network
V2X	Vehicle-to-Everything
SVM	Support Vector Machine
SVDD	Support Vector Data Description
LIMD	Learned Inverse Mapping Detection
UAV	Unmanned Aerial Vehicle
EDFL	Ensemble Dynamic Focus Loss
BCE	Binary Cross-Entropy
F2MD	Framework for Misbehavior Detection in Vehicular Networks
AUC	Area Under the ROC Curve
F1-Score	The harmonic mean of Precision and Recall
FPR	False Positive Rate
G-Mean	Geometric Mean
K-S	Kolmogorov–Smirnov
AUPRC	Area Under the Precision-Recall Curve

## Appendix A

**Table A1:** Summary of Notations.

Symbol	Description
y	Ground-truth label.
p	The model’s predicted probability of the label being 1.
γ	The focusing parameter that controls the strength of dynamic modulation.
$G_{i}$	The *i*-th generator in the feature enhancement module.
$D_{j}$	The *j*-th discriminator in the feature enhancement module.
$L_{real,j}$	The weighted loss for the *j*-th discriminator on real data.
$L_{fake,j}$	The weighted loss for the *j*-th discriminator on fake data.
$L_{D_{j}}$	The total loss for the *j*-th discriminator.
$G_{limd}$	The Distribution Converter in the LIMD module; learns the inverse mapping.
$D_{limd}$	The Latent Space Discriminator in the LIMD module.
x	A data sample vector in the high-dimensional input space.
z	A real sample vector drawn from the target latent distribution.
$G_{limd}\left(x\right)$	A fake latent vector generated by the Distribution Converter from input x.
$S\left(x\right)$	The final anomaly score for an input sample x.
$w_{j}^{\left(k\right)}$	The dynamic modulation weight for the *j*-th discriminator on the *k*-th sample.
α	The hyperparameter balancing the real and fake loss for the discriminator.
